# Supplementary material for: Different photosynthetic adaptation of Zoysia spp. under shading: shade avoidance and shade tolerance response
Source: PeerJ. 2022 Oct 25;10:e14274. doi: 10.7717/peerj.14274 (PMC9615966; doi:10.7717/peerj.14274)
Supplement: Supplemental Information 1 [file peerj-10-14274-s001.docx]

| Accessions | Origin |
| --- | --- |
| ZG-3 | PST-R7ZM, USA |
| Wuhao-1 | Hubei, China |
| WZG99 | Jiangsu, China |
| ZG63 | PI 324184, USA |
| Manila | Hubei, China |
| ZG31 | Grif16485 1 SD, USA |
| Nanling | Jiangsu, China |
| ZG45 | PI 338575, USA |
| WZG55 | Fujian, China |
| WZG59 | Fujian, China |
| ZG66 | USA |
| ZG65 | USA |
| ZG67 | USA |
| WZGF8 | Shandong, China |
| WZG91 | Guangdong, China |
| WZG97 | Guangdong, China |
| ZG64 | USA |
| WZG85 | Guangdong, China |
| ZG48 | PI 338577, USA |
