# Supplementary material for: Different photosynthetic adaptation of Zoysia spp. under shading: shade avoidance and shade tolerance response
Source: PeerJ. 2022 Oct 25;10:e14274. doi: 10.7717/peerj.14274 (PMC9615966; doi:10.7717/peerj.14274)
Supplement: Supplemental Information 2 [file peerj-10-14274-s002.docx]

Y1 = 0.14 X_Plant heights_ + 0.17 X_Chl_ *_a_* + 0.12 X_Chl_ *_b_* + 0.14 X_Caro_ + 0.10 X_Chl_*_a_*_/Chl_*_b_* + 0.21 X_Chls/Caro_ - 0.07 X_Fo_ + 0.18 X_Fm_+ 0.29 X_φPo_ + 0.30 X_ψEo_ + 0.32 X_φEo_ - 0.28 X_ABS/RC_ - 0.24 X_TRo/RC_ + 0.09 X_ETo/RC_ - 0.31 X_DIo/RC_ + 0.34 X_PIABS_ + 0.33 X_PIcs_ + 0.30 X_PItotal_

Y2 = 0.28 X_Plant heights_ + 0.50 X_Chl_ *_a_* + 0.46 X_Chl_ *_b_* + 0.51 X_Caro_ + 0.05 X_Chl_*_a_*_/Chl_*_b_* + 0.21 X_Chls/Caro_ - 0.10X_Fo_ - 0.15 X_Fm_- 0.06 X_φPo_ - 0.06 X_ψEo_ - 0.07X_φEo_ + 0.16 X_ABS/RC_ + 0.17 X_TRo/RC_ + 0.10 X_ETo/RC_ + 0.13 X_DIo/RC_ - 0.11 X_PIABS_ - 0.10 X_PIcs_ -0.10 X_PItotal_

Y3 = 0.13 X_Plant heights_ - 0.03 X_Chl_ *_a_* - 0.24 X_Chl_ *_b_* - 0.03 X_Caro_ + 0.40 X_Chl_*_a_*_/Chl_*_b_* - 0.13 X_Chls/Caro_ + 0.18 X_Fo_ + 0.18 X_Fm_ + 0.01 X_φPo_ + 0.24 X_ψEo_+ 0.21 X_φEo_ + 0.31 X_ABS/RC_ + 0.36 X_TRo/RC_+ 0.55 X_ETo/RC_ + 0.18 X_DIo/RC_+ 0.05 X_PIABS_+ 0.11 X_PIcs_ -0.11 X_PItotal_

Y4 = 0.34 X_Plant heights_ + 0.08 X_Chl_ *_a_*- 0.02 X_Chl_ *_b_* + 0.13 X_Caro_ + 0.22 X_Chl_*_a_*_/Chl_*_b_* - 0.15 X_Chls/Caro_ + 0.63 X_Fo_ + 0.45 X_Fm_- 0.07 X_φPo_ - 0.16 X_ψEo_ - 0.15 X_φEo_ - 0.13 X_ABS/RC_ - 0.16 X_TRo/RC_ - 0.28 X_ETo/RC_ - 0.08 X_DIo/RC_ - 0.10 X_PIABS_ - 0.05 X_PIcs_ - 0.03 X_PItotal_

Y5 = -0.30 X_Plant heights_ - 0.04 X_Chl_ *_a_* + 0.27 X_Chl_ *_b_* - 0.07 X_Caro_ - 0.56 X_Chl_*_a_*_/Chl_*_b_* + 0.34 X_Chls/Caro_ + 0.41 X_Fo_ + 0.33 X_Fm_- 0.12 X_φPo_ + 0.11 X_ψEo_+ 0.07 X_φEo_ + 0.11 X_ABS/RC_ + 0.10 X_TRo/RC_ + 0.20 X_ETo/RC_ + 0.14 X_DIo/RC_ -0.01 X_PIABS_ + 0.03 X_PIcs_+ 0.11 X_PItotal_
