# Supplementary material for: Different photosynthetic adaptation of Zoysia spp. under shading: shade avoidance and shade tolerance response
Source: PeerJ. 2022 Oct 25;10:e14274. doi: 10.7717/peerj.14274 (PMC9615966; doi:10.7717/peerj.14274)
Supplement: Supplemental Information 3 [file peerj-10-14274-s003.docx]

Table S2 Fluorescence parameters derived from the extracted data of the recorded fluorescence transient OJIP of zoysiagrass

| Cultivar | Treatment | Fo | Fm | φPo | ψEo | φEo | ABS/RC | TRo/RC | ETo/RC | DIo/RC | PI_ABS_ | PI_CS_ | PI_total_ |
| --- | --- | --- | --- | --- | --- | --- | --- | --- | --- | --- | --- | --- | --- |
| ZG-3 | light | 0.55±0.017 | 2.86±0.065 | 0.81±0.007 | 0.49±0.021 | 0.39±0.017 | 3.47±0.127 | 2.80±0.104 | 1.36±0.063 | 0.67±0.035 | 1.14±0.127 | 0.63±0.058 | 0.47±0.068 |
|  | 85% shade | 0.58±0.008* | 2.95±0.022 | 0.80±0.002 | 0.47±0.005 | 0.38±0.003 | 3.712±0.073** | 2.99±0.055* | 1.41±0.030 | 0.73±0.019* | 0.98±0.024 | 0.57±0.018 | 0.35±0.009* |
| Wuhao-1 | light | 0.57±0.010 | 2.94±0.051 | 0.81±0.004 | 0.43±0.010 | 0.35±0.010 | 4.02±0.189 | 3.24±0.136 | 1.40±0.029 | 0.78±0.053 | 0.80±0.090 | 0.45±0.048 | 0.30±0.042 |
|  | 85% shade | 0.61±0.013* | 3.05±0.009* | 0.80±0.004 | 0.43±0.016 | 0.35±0.011 | 4.09±0.116 | 3.27±0.076 | 1.42±0.082 | 0.82±0.040 | 0.75±0.021 | 0.46±0.019 | 0.30±0.014 |
| WZG99 | light | 0.58±0.023 | 2.93±0.034 | 0.80±0.006 | 0.44±0.016 | 0.35±0.013 | 3.75±0.099 | 3.00±0.064 | 1.32±0.075 | 0.74±0.040 | 0.85±0.063 | 0.49±0.022 | 0.44±0.072 |
|  | 85% shade | 0.62±0.028 | 2.98±0.042 | 0.79±0.010 | 0.43±0.018 | 0.34±0.018 | 3.97±0.009* | 3.15±0.039* | 1.36±0.072 | 0.82±0.038 | 0.73±0.096 | 0.45±0.044 | 0.32±0.058 |
| ZG63 | light | 0.60±0.037 | 2.78±0.062 | 0.78±0.011 | 0.41±0.026 | 0.32±0.023 | 4.28±0.243 | 3.35±0.145 | 1.39±0.075 | 0.93±0.099 | 0.61±0.124 | 0.36±0.062 | 0.30±0.044 |
|  | 85% shade | 0.63±0.021 | 2.86±0.061 | 0.78±0.011 | 0.47±0.008* | 0.37±0.012* | 4.18±0.383 | 3.26±0.255 | 1.53±0.092 | 0.92±0.130 | 0.77±0.138 | 0.48±0.073 | 0.28±0.027 |
| Manila | light | 0.59±0.016 | 2.68±0.039 | 0.78±0.004 | 0.43±0.006 | 0.34±0.003 | 4.51±0.055 | 3.53±0.057 | 1.51±0.010 | 0.99±0.012 | 0.59±0.010 | 0.35±0.015 | 0.35±0.017 |
|  | 85% shade | 0.62±0.016 | 2.86±0.035** | 0.78±0.003 | 0.47±0.007** | 0.37±0.004** | 4.47±0.079 | 3.50±0.056 | 1.63±0.049* | 0.96±0.027 | 0.71±0.010** | 0.44±0.005** | 0.29±0.040 |
| ZG31 | light | 0.60±0.026 | 2.65±0.031 | 0.77±0.013 | 0.39±0.022 | 0.30±0.022 | 4.51±0.174 | 3.48±0.078 | 1.37±0.060 | 1.03±0.097 | 0.50±0.091 | 0.30±0.044 | 0.24±0.030 |
|  | 85% shade | 0.59±0.017 | 2.75±0.063 | 0.79±0.004 | 0.45±0.011* | 0.36±0.007* | 3.91±0.248* | 3.08±0.212* | 1.40±0.069 | 0.83±0.037* | 0.79±0.059** | 0.46±0.043** | 0.31±0.010* |
| Nanling | light | 0.63±0.044 | 2.86±0.040 | 0.78±0.014 | 0.41±0.011 | 0.32±0.003 | 4.00±0.141 | 3.12±0.114 | 1.29±0.048 | 0.88±0.071 | 0.63±0.035 | 0.39±0.013 | 0.33±0.053 |
|  | 85% shade | 0.64±0.015 | 3.01±0.024** | 0.79±0.004 | 0.45±0.007* | 0.35±0.004 | 3.72±0.168 | 2.93±0.120 | 1.31±0.074 | 0.80±0.049 | 0.80±0.029** | 0.51±0.007** | 0.36±0.026 |
| ZG45 | light | 0.59±0.020 | 2.63±0.100 | 0.78±0.005 | 0.47±0.009 | 0.36±0.009 | 4.07±0.356 | 3.15±0.260 | 1.48±0.114 | 0.91±0.097 | 0.76±0.109 | 0.45±0.062 | 0.38±0.071 |
|  | 85% shade | 0.62±0.052 | 2.84±0.075* | 0.78±0.012 | 0.46±0.013 | 0.36±0.013 | 4.16±0.252 | 3.24±0.183 | 1.49±0.123 | 0.92±0.088 | 0.73±0.080 | 0.45±0.013 | 0.33±0.049 |
| WZG55 | light | 0.57±0.023 | 2.65±0.045 | 0.79±0.006 | 0.39±0.014 | 0.31±0.009 | 4.24±0.074 | 3.33±0.063 | 1.30±0.033 | 0.91±0.029 | 0.55±0.025 | 0.32±0.025 | 0.29±0.026 |
|  | 85% shade | 0.61±0.020 | 3.04±0.026** | 0.80±0.005* | 0.42±0.012* | 0.33±0.008** | 4.02±0.081* | 3.21±0.056* | 1.34±0.054* | 0.80±0.034** | 0.71±0.015** | 0.43±0.015** | 0.28±0.017 |
| WZG59 | light | 0.64±0.017 | 2.58±0.100 | 0.75±0.016 | 0.37±0.029 | 0.28±0.027 | 4.50±0.166 | 3.38±0.054 | 1.26±0.079 | 1.12±0.112 | 0.41±0.093 | 0.26±0.053 | 0.23±0.034 |
|  | 85% shade | 0.61±0.008* | 2.87±0.016** | 0.79±0.003* | 0.45±0.004** | 0.36±0.004** | 4.07±0.036* | 3.21±0.031** | 1.46±0.009 | 0.86±0.016* | 0.76±0.026** | 0.46±0.014** | 0.33±0.023* |
| ZG66 | light | 0.61±0.024 | 2.72±0.058 | 0.78±0.013 | 0.40±0.017 | 0.31±0.019 | 3.86±0.181 | 2.99±0.091 | 1.20±0.026 | 0.87±0.092 | 0.61±0.109 | 0.37±0.055 | 0.40±0.029 |
|  | 85% shade | 0.65±0.018 | 3.09±0.034** | 0.79±0.004 | 0.45±0.012** | 0.36±0.008** | 3.98±0.076 | 3.14±0.048* | 1.42±0.055** | 0.84±0.031 | 0.78±0.027* | 0.51±0.020** | 0.36±0.028 |
| ZG65 | light | 0.62±0.023 | 2.74±0.072 | 0.77±0.010 | 0.44±0.018 | 0.34±0.018 | 4.16±0.242 | 3.21±0.151 | 1.41±0.061 | 0.94±0.094 | 0.65±0.104 | 0.40±0.054 | 0.38±0.063 |
|  | 85% shade | 0.59±0.027 | 3.03±0.046** | 0.81±0.007** | 0.46±0.011 | 0.37±0.006* | 3.84±0.078* | 3.10±0.053 | 1.42±0.035 | 0.74±0.038** | 0.93±0.039** | 0.54±0.029** | 0.37±0.017 |
| ZG67 | light | 0.58±0.022 | 2.43±0.032 | 0.76±0.006 | 0.40±0.007 | 0.31±0.004 | 4.05±0.121 | 3.09±0.116 | 1.24±0.036 | 0.96±0.009 | 0.53±0.011 | 0.31±0.017 | 0.34±0.030 |
|  | 85% shade | 0.61±0.016 | 2.82±0.028** | 0.79±0.007** | 0.45±0.013** | 0.36±0.010** | 4.01±0.148 | 3.25±0.132 | 1.47±0.045** | 0.89±0.031* | 0.74±0.057** | 0.45±0.038** | 0.32±0.030 |
| WZGF8 | light | 0.60±0.014 | 2.76±0.109 | 0.78±0.004 | 0.41±0.017 | 0.32±0.015 | 3.77±0.281 | 2.95±0.220 | 1.21±0.101 | 0.82±0.064 | 0.67±0.088 | 0.40±0.062 | 0.39±0.019 |
|  | 85% shade | 0.62±0.005* | 2.97±0.078* | 0.79±0.005 | 0.46±0.014* | 0.36±0.010** | 3.91±0.302 | 3.09±0.224 | 1.41±0.146 | 0.82±0.080 | 0.82±0.042* | 0.51±0.026* | 0.35±0.027 |
| WZG91 | light | 0.61±0.031 | 2.82±0.057 | 0.78±0.008 | 0.37±0.010 | 0.29±0.005 | 4.09±0.088 | 3.21±0.039 | 1.19±0.042 | 0.88±0.050 | 0.52±0.019 | 0.32±0.007 | 0.31±0.025 |
|  | 85% shade | 0.63±0.016 | 3.10±0.208** | 0.80±0.006* | 0.43±0.009** | 0.34±0.008** | 3.98±0.158 | 3.18±0.123 | 1.36±0.082* | 0.80±0.043 | 0.74±0.032** | 0.46±0.009** | 0.33±0.026 |
| WZG97 | light | 0.54±0.024 | 2.66±0.015 | 0.80±0.009 | 0.40±0.015 | 0.32±0.008 | 4.22±0.106 | 3.36±0.122 | 1.34±0.011 | 0.85±0.016 | 0.62±0.020 | 0.33±0.025 | 0.32±0.017 |
|  | 85% shade | 0.60±0.014** | 2.93±0.055** | 0.80±0.007 | 0.44±0.016* | 0.35±0.010** | 4.27±0.137 | 3.39±0.090 | 1.49±0.072* | 0.87±0.052 | 0.72±0.037* | 0.43±0.018** | 0.26±0.021* |
| ZG64 | light | 0.57±0.029 | 2.80±0.095 | 0.80±0.015 | 0.43±0.011 | 0.34±0.015 | 3.47±0.173 | 2.76±0.132 | 1.19±0.070 | 0.70±0.070 | 0.87±0.141 | 0.49±0.062 | 0.44±0.046 |
|  | 85% shade | 0.63±0.027 | 3.05±0.034* | 0.79±0.007 | 0.43±0.014 | 0.34±0.012 | 3.90±0.010* | 3.10±0.019* | 1.34±0.045* | 0.80±0.029 | 0.76±0.055 | 0.48±0.032 | 0.26±0.048** |
| WZG85 | light | 0.58±0.039 | 2.66±0.045 | 0.78±0.015 | 0.40±0.028 | 0.31±0.028 | 4.56±0.227 | 3.56±0.147 | 1.41±0.113 | 1.00±0.107 | 0.52±0.122 | 0.30±0.051 | 0.25±0.059 |
|  | 85% shade | 0.57±0.031 | 2.77±0.024* | 0.79±0.010 | 0.43±0.007 | 0.34±0.003 | 4.28±0.068 | 3.39±0.088 | 1.45±0.030 | 0.88±0.035 | 0.67±0.016 | 0.38±0.012 | 0.24±0.005 |
| ZG48 | light | 0.61±0.006 | 2.47±0.042 | 0.75±0.002 | 0.34±0.023 | 0.26±0.018 | 4.55±0.185 | 3.43±0.144 | 1.16±0.129 | 1.12±0.042 | 0.35±0.024 | 0.21±0.014 | 0.21±0.023 |
|  | 85% shade | 0.62±0.014 | 2.93±0.068** | 0.80±0.009** | 0.45±0.041* | 0.35±0.029** | 3.98±0.055** | 3.14±0.064* | 1.40±0.124 | 0.84±0.035** | 0.76±0.091** | 0.47±0.066** | 0.29±0.026* |

Data represent means of three replicates, and asterisks indicate significant differences at P<0.05 (*), P<0.01 (**).
