# Supplementary material for: Different photosynthetic adaptation of Zoysia spp. under shading: shade avoidance and shade tolerance response
Source: PeerJ. 2022 Oct 25;10:e14274. doi: 10.7717/peerj.14274 (PMC9615966; doi:10.7717/peerj.14274)
Supplement: Supplemental Information 4 [file peerj-10-14274-s004.docx]

| Accessions | Plant height | Chl*_a_* | Chl*_b_* | Caro | Chl*a*/Chl*b* | Chls/Caro | Fo | Fm | φPo | ψEo | φEo | ABS/RC | TRo/RC | ETo/RC | DIo/RC | PI_ABS_ | PI_CS_ | PI_total_ |
| --- | --- | --- | --- | --- | --- | --- | --- | --- | --- | --- | --- | --- | --- | --- | --- | --- | --- | --- |
| ZG-3 | 0.92±0.058 | 1.07±0.088 | 1.07±0.049 | 1.03±0.054 | 1.00±0.040 | 1.03±0.028 | 1.05±0.029 | 1.03±0.024 | 1.00±0.012 | 0.97±0.056 | 0.97±0.056 | 1.07±0.012 | 1.07±0.010 | 1.04±0.051 | 1.09±0.063 | 0.86±0.117 | 0.91±0.118 | 0.75±0.130 |
| Wuhao-1 | 1.05±0.118 | 1.29±0.022 | 1.43±0.039 | 1.24±0.002 | 0.90±0.026 | 1.05±0.015 | 1.07±0.031 | 1.04±0.018 | 0.99±0.005 | 1.00±0.057 | 0.99±0.058 | 1.02±0.036 | 1.01±0.032 | 1.01±0.039 | 1.05±0.056 | 0.94±0.136 | 1.01±0.148 | 1.00±0.204 |
| WZG99 | 1.28±0.364 | 1.49±0.154 | 1.56±0.115 | 1.47±0.256 | 0.96±0.176 | 1.01±0.085 | 1.06±0.031 | 1.02±0.025 | 0.99±0.005 | 0.98±0.050 | 0.97±0.054 | 1.06±0.029 | 1.05±0.034 | 1.03±0.084 | 1.10±0.013 | 0.87±0.073 | 0.92±0.070 | 0.73±0.031 |
| ZG63 | 0.80±0.132 | 0.90±0.018 | 0.87±0.076 | 0.88±0.057 | 1.03±0.089 | 1.02±0.049 | 1.04±0.051 | 1.03±0.0122 | 1.00±0.012 | 1.14±0.054 | 1.13±0.044 | 0.98±0.081 | 0.97±0.068 | 1.11±0.123 | 0.99±0.124 | 1.26±0.131 | 1.32±0.077 | 0.93±0.058 |
| Manila | 1.10±0.213 | 1.16±0.058 | 1.08±0.120 | 1.09±0.050 | 1.07±0.151 | 1.06±0.012 | 1.05±0.004 | 1.07±0.006 | 1.00±0.001 | 1.09±0.015 | 1.09±0.015 | 0.99±0.020 | 0.99±0.021 | 1.08±0.026 | 0.97±0.016 | 1.20±0.036 | 1.26±0.041 | 0.82±0.148 |
| ZG31 | 0.96±0.083 | 1.29±0.0327 | 1.33±0.087 | 1.22±0.045 | 0.97±0.060 | 1.06±0.013 | 0.97±0.016 | 1.04±0.029 | 1.02±0.012 | 1.15±0.094 | 1.17±0.108 | 0.87±0.073 | 0.88±0.068 | 1.02±0.020 | 0.81±0.091 | 1.59±0.446 | 1.56±0.405 | 1.31±0.169 |
| Nanling | 1.09±0.152 | 1.412±0.0573 | 1.54±0.122 | 1.35±0.051 | 0.92±0.039 | 1.06±0.018 | 1.02±0.085 | 1.05±0.023 | 1.01±0.022 | 1.08±0.041 | 1.09±0.019 | 0.93±0.076 | 0.94±0.070 | 1.02±0.097 | 0.91±0.120 | 1.27±0.113 | 1.30±0.057 | 1.10±0.211 |
| ZG45 | 1.05±0.099 | 1.19±0.092 | 1.15±0.123 | 1.16±0.094 | 1.04±0.034 | 1.02±0.006 | 1.06±0.137 | 1.08±0.073 | 1.01±0.020 | 0.98±0.017 | 0.98±0.016 | 1.02±0.173 | 1.03±0.161 | 1.01±0.175 | 1.00±0.221 | 0.96±0.196 | 1.01±0.142 | 0.89±0.278 |
| WZG55 | 1.20±0.182 | 1.50±0.071 | 1.50±0.207 | 1.43±0.069 | 1.00±0.092 | 1.06±0.011 | 1.07±0.013 | 1.15±0.008 | 1.02±0.002 | 1.07±0.039 | 1.09±0.039 | 0.95±0.009 | 0.97±0.009 | 1.03±0.033 | 0.88±0.012 | 1.29±0.082 | 1.38±0.100 | 0.96±0.039 |
| WZG59 | 1.16±0.172 | 2.03±0.108 | 2.01±0.181 | 1.76±0.120 | 1.01±0.096 | 1.15±0.025 | 0.95±0.026 | 1.11±0.079 | 1.05±0.024 | 1.22±0.110 | 1.27±0.142 | 0.90±0.041 | 0.95±0.023 | 1.16±0.075 | 0.77±0.088 | 1.86±0.542 | 1.78±0.472 | 1.44±0.322 |
| ZG66 | 1.22±0.197 | 1.58±0.207 | 1.21±0.069 | 1.48±0.198 | 1.31±0.151 | 1.04±0.016 | 1.06±0.057 | 1.14±0.018 | 1.02±0.018 | 1.13±0.024 | 1.15±0.045 | 1.03±0.058 | 1.05±0.042 | 1.19±0.023 | 0.96±0.109 | 1.29±0.223 | 1.37±0.166 | 0.91±0.091 |
| ZG65 | 1.25±0.036 | 1.31±0.068 | 1.17±0.124 | 1.21±0.067 | 1.12±0.063 | 1.07±0.006 | 0.95±0.048 | 1.11±0.018 | 1.04±0.019 | 1.05±0.053 | 1.09±0.072 | 0.92±0.089 | 0.96±0.075 | 1.01±0.058 | 0.79±0.127 | 1.42±0.386 | 1.35±0.292 | 0.97±0.197 |
| ZG67 | 1.14±0.110 | 1.71±0.100 | 1.66±0.168 | 1.58±0.096 | 1.03±0.053 | 1.08±0.007 | 1.05±0.053 | 1.16±0.021 | 1.03±0.012 | 1.14±0.053 | 1.17±0.052 | 1.02±0.022 | 1.05±0.030 | 1.19±0.032 | 0.93±0.032 | 1.39±0.137 | 1.46±0.171 | 0.94±0.051 |
| WZGF8 | 1.46±0.202 | 1.46±0.044 | 1.39±0.027 | 1.37±0.027 | 1.05±0.027 | 1.06±0.012 | 1.04±0.031 | 1.08±0.074 | 1.01±0.010 | 1.12±0.054 | 1.13±0.065 | 1.04±0.092 | 1.05±0.093 | 1.17±0.132 | 0.99±0.099 | 1.23±0.181 | 1.27±0.228 | 0.91±0.013 |
| WZG91 | 1.31±0.184 | 1.32±0.069 | 1.25±0.119 | 1.25±0.073 | 1.06±0.102 | 1.04±0.021 | 1.03±0.076 | 1.10±0.023 | 1.02±0.016 | 1.15±0.022 | 1.17±0.026 | 0.97±0.002 | 0.99±0.015 | 1.14±0.026 | 0.91±0.054 | 1.42±0.116 | 1.46±0.019 | 1.08±0.098 |
| WZG97 | 1.52±0.045 | 2.11±0.070 | 1.81±0.144 | 1.92±0.053 | 1.17±0.114 | 1.08±0.007 | 1.11±0.055 | 1.10±0.021 | 1.00±0.019 | 1.10±0.083 | 1.10±0.061 | 1.01±0.035 | 1.01±0.038 | 1.11±0.071 | 1.02±0.090 | 1.157±0.0645 | 1.28±0.120 | 0.83±0.052 |
| ZG64 | 0.72±0.062 | 1.00±0.0315 | 0.98±0.132 | 0.98±0.036 | 1.02±0.139 | 1.01±0.003 | 1.11±0.083 | 1.09±0.027 | 1.00±0.027 | 1.01±0.031 | 1.00±0.051 | 1.13±0.057 | 1.12±0.051 | 1.13±0.035 | 1.14±0.164 | 0.88±0.174 | 0.98±0.146 | 0.59±0.171 |
| WZG85 | 1.54±0.140 | 1.62±0.082 | 1.46±0.170 | 1.46±0.071 | 1.11±0.078 | 1.09±0.011 | 0.98±0.101 | 1.04±0.009 | 1.02±0.030 | 1.08±0.074 | 1.09±0.101 | 0.94±0.041 | 0.95±0.034 | 1.03±0.100 | 0.88±0.116 | 1.28±0.338 | 1.27±0.193 | 0.94±0.234 |
| ZG48 | 1.53±0.073 | 1.35±0.057 | 1.12±0.122 | 1.23±0.051 | 1.20±0.039 | 1.07±0.018 | 1.02±0.085 | 1.19±0.023 | 1.05±0.022 | 1.32±0.041 | 1.38±0.019 | 0.88±0.076 | 0.91±0.070 | 1.20±0.097 | 0.75±0.120 | 2.19±0.113 | 2.24±0.057 | 1.41±0.211 |
